# Soft-Lithography of Polyacrylamide Hydrogels Using Microstructured Templates: Towards Controlled Cell Populations on Biointerfaces

**DOI:** 10.3390/ma13071586

**Published:** 2020-03-30

**Authors:** Andrés Díaz Lantada, Noelia Mazarío Picazo, Markus Guttmann, Markus Wissmann, Marc Schneider, Matthias Worgull, Stefan Hengsbach, Florian Rupp, Klaus Bade, Gustavo R. Plaza

**Affiliations:** 1 Product Development Laboratory, Mechanical Engineering Department, Universidad Politécnica de Madrid, c/José Gutiérrez Abascal 2, 28006 Madrid, Spain; noelia.mazario@gmail.com; 2 Centre for Biomedical Technology, Universidad Politécnica de Madrid, Parque Científico y Tecnológico de la UPM, Crta. M40, km. 38, 28223 Pozuelo de Alarcón, Madrid, Spain; gustavo.plaza@upm.es; 3 Institute of Microstructure Technology, Karlsruhe Institute of Technology, Hermann-von-Helmholtz Platz 1, 76344 Eggenstein-Leopoldshafen, Germany; markus.guttmann@kit.edu (M.G.); markus.wissmann@kit.edu (M.W.); marc.schneider2@kit.edu (M.S.); matthias.worgull@kit.edu (M.W.); stefan.hengsbach@kit.edu (S.H.); florian.rupp@kit.edu (F.R.); klaus.bade@kit.edu (K.B.)

**Keywords:** polymer microfabrication, polyacrylamide hydrogels, hot-embossing, soft-lithography, biointerfaces, surface patterning

## Abstract

Polyacrylamide hydrogels are interesting materials for studying cells and cell–material interactions, thanks to the possibility of precisely adjusting their stiffness, shear modulus and porosity during synthesis, and to the feasibility of processing and manufacturing them towards structures and devices with controlled morphology and topography. In this study a novel approach, related to the processing of polyacrylamide hydrogels using soft-lithography and employing microstructured templates, is presented. The main novelty relies on the design and manufacturing processes used for achieving the microstructured templates, which are transferred by soft-lithography, with remarkable level of detail, to the polyacrylamide hydrogels. The conceived process is demonstrated by patterning polyacrylamide substrates with a set of vascular-like and parenchymal-like textures, for controlling cell populations. Final culture of amoeboid cells, whose dynamics is affected by the polyacrylamide patterns, provides a preliminary validation of the described strategy and helps to discuss its potentials.

## 1. Introduction

Hydrogels play a fundamental role in areas such as tissue engineering, biofabrication, biotechnology and medical devices, including the development of innovative labs- and organs-on-chips and three-dimensional cell-laden microstructures, as they are able to provide cells with tunable and biomimetic extracellular matrices to replicate and study a wide set of physiological functions [1,2,3]. Their use leads to studying cells, cell–material interactions, physiological processes and disease in 3D and 4D microenvironments, which represent human/animal nature more adequately, than when resorting to conventional cell culture processes using 2D Petri dishes.

Within hydrogels employed for medical applications, the family of polyacrylamide hydrogels has been used since the 1980s [4] until nowadays [5] for the development of drug-eluting structures and gel-coated medical devices. Polyacrylamide hydrogels also stand out for being typically biocompatible, clear and non-fluorescent, which facilitates the microscopic visualization of cells and cellular processes [6,7].

The mentioned properties, linked to the possibility of controlling their stiffness, porosity and shear modulus, among other parameters, during their synthesis, have made polyacrylamide hydrogels very valuable for studying cell–material interactions and the effects of stiffness on cell morphology and mechanics [6,7].

Mechanisms of both prokaryotes and eukaryotes have been studied with the support of hydrogels, in general, and of polyacrylamide gels, in particular, in connection to better understanding disease and healing processes and the effects of cell–material interactions, substrate morphology and surface chemistry on gene expression, cell dynamics and biofilm growth [6,7].

In the last decade, these polyacrylamide hydrogels have shown benefits for cellular studies, in terms of control of polymeric chain length and related precise tuning of physico-chemical properties, as compared with more traditional substrate materials including: agar, gellan, xanthan gum or alginate, among others [7]. As a consequence of their singular characteristics, they have been progressively incorporated to the vast materials portfolio employed in biophysical studies, tissue engineering, biofabrication, biotechnology and biomedical engineering in general.

Lately, in order to promote more precise cell–material interactions and more specific and efficient studies using hydrogels and polyacrylamide gels, improvements to their syntheses processes have been combined with enhanced processing techniques for achieving micrometric (and in some cases even nanometric) control of their morphological and topographical features. These improvements are enabling interactions at the single-cell level and helping to control cell populations and their behavior (and fate) upon polyacrylamide biointerfaces. Among the more interesting processing techniques, developed for controlling polyacrylamide gel morphology, it is important to highlight the use of three-dimensional (micro)molding processes [8], in some cases containing living microorganisms within the molds and created structures [9], and the employment of patterning techniques, typically based on soft-lithography using microfabricated templates [10]. These sets of processes may be also synergistically combined for achieving three-dimensional structures with patterned surfaces.

More recently, other techniques have focused on the creation of multi-scale features and, for example, parylene C films have been used as masks for directly copolymerizing proteins on polyacrylamide gels and for studying the effect of patterned polyacrylamide on the morphology and orientation of cultured cells [11]. Nanoreplica molding using silicon molds and benefiting from micromanufacturing technologies common from the electronic industry has been applied to transferring patterns to polyacrylamide gels and validated as single cell array and proposed for lab-on-a-chip and biosensing applications [12]. Multi-scale strategies, based on the micromolding of electrospun nanofibrous mats and validated through the production gelatin methacrylate scaffolds with micro and nano topographical features [13], could possibly be adapted for processing polyacrylamide hydrogels. New horizons are opened up by the synthesis of electrically conductive [14] and stimuli-responsive [15] hydrogels and by the application of innovative biofabrication processes [16], including high-resolution bioprinting [17], which may increase the functionality and complexity of the hydrogel structures and promote novel medical applications in different areas, once challenges linked to precision, stability and repeatability are solved.

In this study a novel approach, related to the processing of polyacrylamide hydrogels using soft-lithography and employing microstructured templates, is presented and analyzed. The novelty relies on the templates used: Their multi-scale microstructures are designed by means of 3D computational modeling and rapid master models are created by direct laser writing of photopolymers. PVD metallization and electroplating transforms the master models in mold inserts, for systematic cost- and time-effective replication employing hot-embossing (or compression molding). The templates obtained through hot-embossing of thermoplastic films are transferred by soft-lithography, with remarkable level of detail, to the polyacrylamide hydrogels. Details in the 1–10 μm range, which allow for single-cell manipulation, are achieved. 

The conceived process is demonstrated by patterning polyacrylamide substrates with a set of vascular-like and parenchymal-like textures, which are conceived as potential functional layers or biointerfaces of lab- and organ-on-a-chip devices for controlling cell populations, as also discussed in the discussion and future proposals section of this paper.

Final culture of amoeboid cells, whose dynamics is affected by the polyacrylamide patterns, as they arrange themselves forming clusters according to the designed geometries, provides a preliminary validation of the described strategy and helps to discuss its potentials, towards more complex and biomimetic co-culture systems and cell cultivation set-ups.

## 2. Materials and Methods

### 2.1. Computer-Aided Design of Microstructured Surfaces

Multi-scale topographies were designed by adding them to desired zones of planar surfaces, thus creating design-controlled transitions of roughness, following a previously described process developed by our team [18] with some modifications. The process uses mathematical functions for the generation of height matrixes, which stores the information of a height function z(x,y) evaluated in the set of points of a grid defined in the x-y plane, and post-processes the generated matrixes using state-of-the-art computer-aided design (CAD) software. Summarizing, a mathematical model is evaluated above a grid, in accordance with the precision of the additive manufacturing process, to be employed for the manufacture of master models.

The multi-scale surface may be obtained as sum of the micro/nanotextures and, in our case, are stored in form of MATLAB (The Mathworks Inc., MA, United States) surface or surfaces. Once the surfaces are defined, their geometrical features can be stored in the form of a [X, Y, Z] matrixes for further processing or for conversion into .stl (standard tessellation language) or any CAD format apt for exchange, so that the surface can be subject to additional design operations employing CAD software. For this research we used, as mathematical model, the absolute values of a sum of sinusoidal functions, of different amplitude and frequency, so as to create positive microbumps (that will turn out to be microwells upon the polyacrylamide gel) with a height of 10 mm and occupying regions of 10 × 10 µm^2^.

The microbumps designed have rough surfaces, thanks to the incorporation of smaller bumps of around 2 × 2 × 2 µm^3^, which try to mimic the surface topography of interesting plant leaves with special surfaces and contact properties [19]. The transition between planar zones and microbumps was conceived for creating differential textures capable of interacting at a cellular level and of controlling cell populations upon the obtained templates. In fact, the microbumps were designed to have a size similar to that of the amoeboid cells that will be used for the cell culture and validation experiments. 

**Figure 1:**
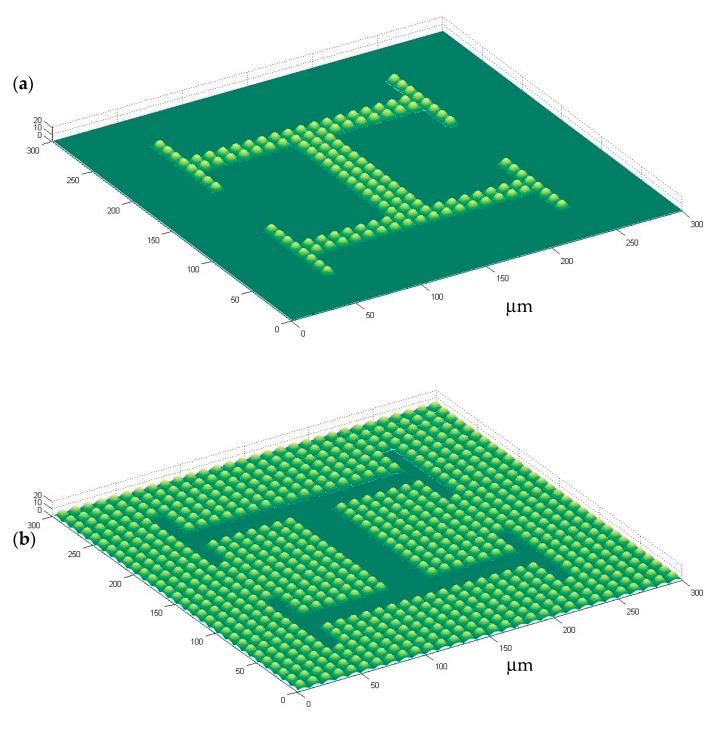
Computer-aided design of microstructured surfaces showing both positive and negative alternatives: (**a**) Microtextured vascular-like region upon a plane; (**b**) planar vascular-like region surrounded by microtexture. Scale in micrometers.

In this research we opted for creating patterns imitating microvasculatures, using a checkboard template (as can be seen in Figure 1). In some cases the microbumps form multi-branched “H-like” vascular textures, in other cases the microbumps surround planar “H-like” zones. It is interesting to note that the positive microbumps were transferred by soft-lithography to the polyacrylamide gels, as explained below, which is expected to create a set of microwells upon the gel surface, capable of trapping the cultured cells and of fixing them, one-by-one, to desired positions.

### 2.2. Production of Hydrogel Templates with Microstructured Surfaces

The production of polyacrylamide gel microstructured surfaces or templates is based on a combination of: (1) High-precision additive photopolymerization, for the generation of the master models; (2) metallization or electroplating, for the rapid manufacturing of compression molding inserts; (3) hot-embossing, for the cost-effective creation of template copies employing thermoplastic polymers; and (4) transfer of patterns to the polyacrylamide gel by soft-lithography. 

The different manufacturing processes and the cell culture experiments employed for validation purposes are detailed in the following subsections.

#### 2.2.1. 3D Direct Laser Writing of Master Models with Design-Controlled Features

Creation of the original models (automatically working with the CAD files) was done using 3D direct laser writing (3D-DLW), a highly precise additive manufacturing technique. In this study, the Photonic Professional System from NanoScribe GmbH was employed. In short, the 3D DLW technology operates a bit differently from common 3D printing techniques, which work on a layer-by-layer fashion.

Here, MATLAB (The Mathworks Inc., MA, United States) was employed to generate the layout data and the data input files (stl format) that can be used directly by the Nanoscribe conversion software. 3D paths were defined for polymerizing using ultra short laser pulses.

The NanoScribe machine employs a laser source from Toptica (Femto Fiber pro NIR) with a wavelength of 780 nm. The machine setup combines a laser and an inverted microscope, which is synchronized and controlled by a computer. The beam is guided through an oil-immersion microscope objective (Zeiss, 63X, NA 1.4) and focused into a resist (acrylate based Ip-DIP, Nanoscribe), placed upon a glass substrate which is rinsed with 2-Propanol. In order to improve the adhesion of the created structures the substrate is heated to 120 °C for 10 min. The mounted glass substrate is displaced by motor stages (Physics Instruments M511.HD1) and a piezo drive (Physics Instruments P-562.3CD) is employed for z-travel. 

For this research study, the microstructured surfaces were obtained by writing tiles (300 × 300 µm for each square of the checkerboard) with the help of the galvo scan unit. Such tiles were stitched together, in order to structure a larger area of 1.8 mm × 1.8 mm. The galvo scan unit scans the laser beam within 150 × 150 µm fields. The writing was performed using a slicing distance of 50 nm (in the z-direction) and employing a hatching distance of 75 nm (within the XY plane). The scan speed of the galvo scan unit was adjusted to 25,000 µm/s. With these parameters and the described writing strategy, a field of 300 × 300 µm was obtained in around 330 s. Final development of the microstructured surfaces was done by washing in PGMEA (propylene glycol methyl ether acetate) two times for 20 min. A third washing step with 2-Propanol for 10 min was also utilized.

#### 2.2.2. Electroplating of Master Models as Compression Molding Tools

The microstructured polymeric surfaces (obtained by 3D DLW on the 25 × 25 mm^2^ glass coverslip) needed to be directly transferred or converted into a metallic mold insert or cavity by electroforming, for which a previously developed process at IMT-KIT with modifications was employed [20,21]. First of all, the glass master with the 3D direct laser written surfaces was glued into a desired cavity of an 8 mm thick copper substrate. Employing an evaporation process, both master and substrate were coated with superimposed layers of chromium (7 nm width) and gold (40 nm width). The chromium layer was employed as adhesive layer and the gold layer helps to achieve a conductive plating base. The metallic layers promoted a precise galvanic metal deposition throughout the microtextured surfaces. To this end, the copper substrate was attached to a commercial plating holder and immersed into galvanic bath. The nickel electroplating system, which works with a boric acid containing (chloride-free) nickel sulphamate electrolyte (T = 52 °C and pH = 3.4 to 3.6), was developed especially for the nickel electroforming of microstructures at IMT-KIT, as previously detailed [22]. The use of this electrolyte leads to remarkably matt, nearly stress-free, thick nickel layers up to 10 mm and without any relevant warpage [23].

In order to achieve an exact electroplating of the microstructured or textured surfaces, a slow growing process was employed: For such slow growth, the current density was adjusted to 0.1 A/dm^2^ at the beginning of the electroplating and was progressively increased up to 1.5 A/dm^2^. Electroforming continued until a nickel layer with a thickness of around 4 mm was obtained. To promote the adhesion of the thick nickel block and to avoid a lift-off during such a long plating time (larger than 2 weeks), the copper substrate was equipped with six threaded holes for toothing. This electroplating process led to a very stiff and homogenous metal block and to a very uniform thickness, which was necessary for supporting the mechanical and thermal stresses that take place in the subsequent hot-embossing procedure.

The achieved electroplated nickel block, with a nice flat surface and without any blowholes or dendrites, was then separated from the copper substrate and mechanically processed (using wire EDM) to obtain the specified external dimensions (32 × 32 × 2.5 mm), which allowed for an adequate fitting into the available hot-embossing tool. 

**Figure 2:**
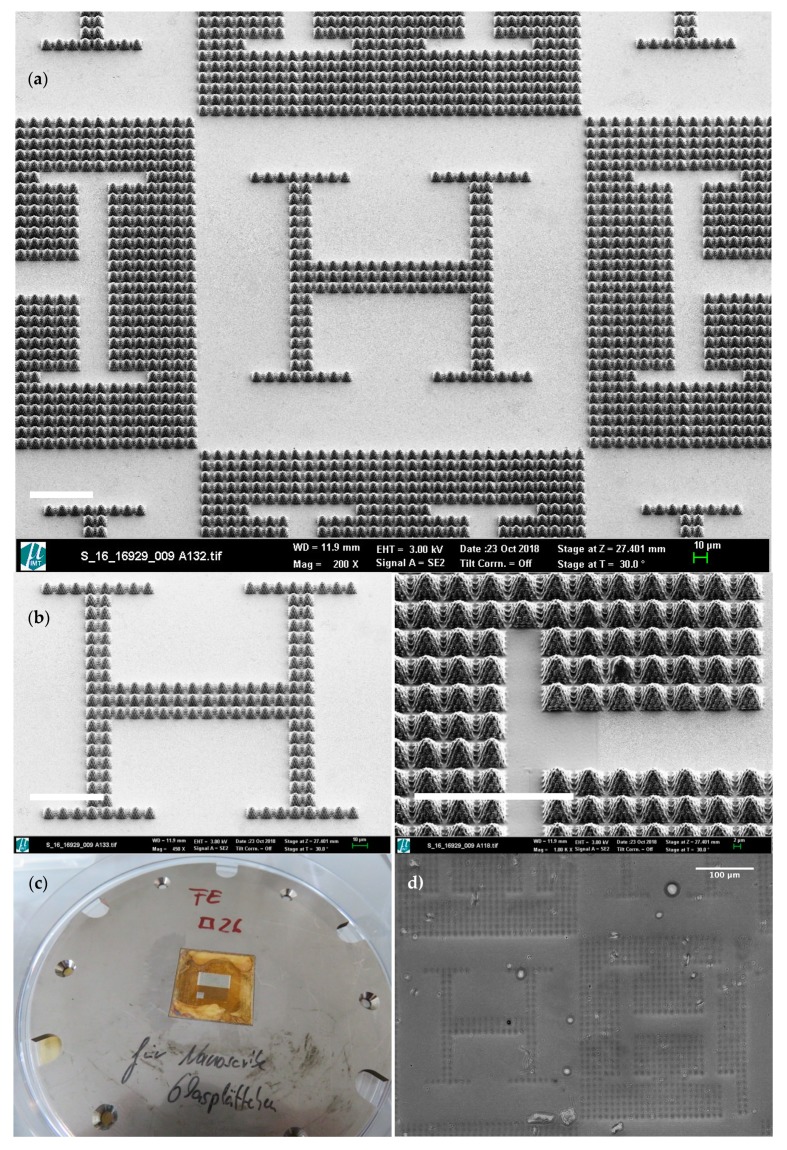
(**a**) Direct laser written microstructures as master models (SEM). (**b**) Detailed views of vascular-like microstructures with different magnification (SEM). (**c**) Mold insert obtained after electroplating and mounted in the hot-embossing tool (digital camera). (**d**) Pattern transferred to the polyacrylamide gel (stereoscopic microscope). White scale bars in a and b = 100 µm.

The DLW glass substrate was removed from the mold insert cavity by using a wet-chemical process and the DLW resist was stripped using a plasma treatment. Structure characterization by scanning electron microscopy (SEM, Carl Zeiss AG, Oberkochen, Germany), as quality control (see Figure 2 for details), completed the nickel mold or embossing tool fabrication.

We would like to highlight that the use of electroforming led to a direct galvanic replication of all relevant structural details (in different scales ranging from nano to micro details) of the master model structures. Furthermore, angled side walls and wavy surfaces could also be transferred from the original resin structures of the master to the metallic tool or mold. The mold was finally mounted and adjusted into the hot-embossing tool.

#### 2.2.3. Hot-Embossing of Microstructured Surfaces

Polymeric copies of the vascular-like textures were manufactured by hot embossing of standard poly (methyl methacrylate) (PMMA) foils using the previously described nickel mold. PMMA is a relevant thermoplastic polymer for the biomedical industry and adequate for cell culture applications and was employed here for soft-lithography of the polyacrylamide gels (see Section 2.3). PMMA foils with a thickness of around 500 µm were placed between the mold insert and a polished steel plate, so as to guarantee a smooth back surface of the replicated templates. The hot-embossing process was carried out with the support of a modified tensile testing machine (Zwick “Retro line”), similar to an embossing system “Jenoptik HEX03” and using the following process parameters: A hot-embossing temperature of 165 °C, a hot-embossing force of 18 kN and a demolding temperature of 95 °C.

### 2.3. Synthesis and Soft-Lithography of Microstructured Polyacrylamide Hydrogels

The materials listed in Table 1 were used in the synthesis of polyacrylamide (PAA) gel. In addition to them, other typical laboratory materials were employed such as pipettes, Falcon tubes, Eppendorf tubes, precision balances, pipettes, so as to work in laminar flow cabinet with cells and Petri dishes of various sizes, among others.

The stiffness, geometry and roughness of polyacrylamide gel are properties that influence the migration process and cell adhesion and may also have an influence on viable patterning by soft-lithography. Stiffness of PAA gels can be controlled by simple changes of polymer precursor concentration [23,24]. Therefore, two different stiffnesses were studied—5% polyacrylamide gels have the lowest stiffness, around 1.5 kPa, while 7% polyacrylamide gels are stiffer, with values in the order of 15 kPa, according to the information from previous studies [25,26].

The first step of the synthesis protocol takes approximately one hour. It consists of the activation of 30 mm diameter glass coverslips on the bottom of a plastic plate to improve the covalent adhesion of PAA gel to the glass coverslips. Some of the chemicals listed in Table 1 were used: 

Firstly, 100 µL of NaOH (0.1 M) were added to the bottom of the plate creating a circular surface after waiting 20 min During the waiting time, a solution of 0.5% glutaraldehyde in PBS 1× was prepared, which was required to carry out the last step of the glass activation. 

To do this, the glutaraldehyde was removed from the freezer. Once defrosted, the solution was prepared in an Eppendorf by adding 1 mL of PBS 1× and 20 µL of GA. After 20 min, the NaOH was removed by cleaning the glass with paper. Then, 15 µL of 3-Aminopropyl) triethoxysilane (APTES) was added with a 1 mL syringe in the same region where the NaOH had been previously deposited. Since APTES is a toxic compound, due to the vapors it releases, this step of the protocol should be carried out in the laboratory fume hood and all waste in contact with this compound should be deposited in the APTES container inside the fume hood. After 5 min, the plates were washed with distilled water and cleaned with paper. Finally, the previously-prepared 0.5% GA solution in PBS 1× was dropped over the plate. The duration of this step is 30 min. Once the waiting time was over, the plate was cleaned with distilled for finishing the glass activation process.

For the second process, 15 mL Falcon tubes were needed to synthesize the two types of polyacrylamide gels. The chemicals listed in Table 2 were used in the quantities detailed. This step was done without stepped waiting processes because TEMED catalyst acts rapidly producing the polymerization of polyacrylamide. The greater the stiffness of the gel, the sooner it gels. Once all reagents were mixed in the Falcon tubes, 150 µL were taken and gently deposited upon different microtextured replicas, so as to replicate the surface structures by soft-lithography. After one hour, the gel was removed, by gentle detachment, from the microstructured surfaces with the help of tweezers.

### 2.4. Cell Culture Experiments upon Polyacrylamide Hydrogel Biointerfaces

*Dictyostelium discoideum* (Amoebozoa, infraphylum Mycetozoa) cells were used for the cell-movement and cell–material interaction experiments. Specifically, in the present study the strain or cell line of *Dictyostelium discoideum* used in the experiments was AX2 [27], provided by Dictybase (Strain-DBS0235534-X2-214, dictybase.org, Northwestern University). They are cells cultivated in an axenic medium and have slower growth rates than cells found in nature, with a doubling time of approximately 8–12 h, instead of the 4 h found in natural environments. Additionally, the doubling time depends on temperature and culture medium [28]. 

When *Dictyostelium discoideum* cells are cultured in the laboratory with an axenic medium, as is the case with the employed HL5-C medium, it is necessary to renew the culture plate medium every 3 or 4 days to keep it clean. It is also a good recommendation to divide the culture plates when the cell density is approximately 4 × 10^5^ cells/mL to ensure a healthy cell population as prolonged culture will lead to the accumulation of undesirable mutations. 

Once the polyacrylamide gel with the corresponding geometry was prepared, *Dictyostelium discoideum* cells were deposited on the surface of the gel. Before depositing the cells, it was necessary to sterilize the gel leaving it in laminar flow cabinet for 30 min with ultraviolet light. Then, 1 mL of cells were extracted from the Petri dish and deposited on the plate containing the gel with the corresponding pattern or surface microstructure. Cells were cultured with HL5-C medium, which is an axenic medium suitable for cultivation of *Dictyostelium discoideum* cells and whose composition listed below in Table 3. It was prepared as a solution of 26.65 g of commercial powder medium (Formedium) per 1 L of distilled water. In this case, a solution of 500 mL of water was prepared, in which 13.27 g of the powdered medium were dissolved. After the solution was prepared, it was agitated in the magnetic stirrer to obtain a homogeneous mixture. 

Later, the prepared medium was sterilized in an autoclave. An autoclaving time of 20 min and a drying time of 10 min were employed. Finally, antibiotics (penicillin and streptomycin in an amount of 10 mL/L) were added to avoid contamination.

### 2.5. Cell Visualization and Tracking

Visualization of *Dictyostelium discoideum* cells was performed with the support of two microscopes: An optical microscope MEIJI TC5400 using its phase contrast mode for visualizing cells without needing staining and a Leica EZ4HD stereoscopic microscope for geometric characterization of the polyacrylamide hydrogels. In order to analyze cell trajectories on the different substrates studied, CellTracker, executed with MATLAB (The Mathworks Inc., MA, United States), was also used. CellTracker is a software resource that processes images in order to analyze cell migration processes [29,30].

This program has three monitoring modalities available. Automatic tracking, which is a combination of template matching and a tracking algorithm, is one of the working modes. Semi-automatic tracking, which allows selection of the desired cells for tracking, relies on an algorithm that defines a specific template for each selected cell and looks for the best match in the consecutive tables. An adaptive template method is used to handle slight cell deformations over time. The third option is manual tracking, which is based on defining the position of the cell in each frame of each consecutive image. Automatic tracking is the best option for applications where cell detection is relatively straightforward. Semi-automatic tracking is the fastest mode and represents a good compensation between cell detection and tracking accuracy. However, as expected, the most accurate solution is manual tracking. 

In conclusion, CellTracker is a versatile tool capable of tracking cells in different scenarios that combines precision and ease of use.

In this study, once microscopy .tiff files were created, with the support of ImageJ software, while CellTracker program was employed for studying cell motion. Before starting the analysis of the trajectories, intensity and contrast of images were adjusted, in order to make the study easier later. Normally, the analysis is performed in semi-automatic mode, except in cases where the cells move closely together, in which cases manual mode is employed. 

After the cell trajectories were analyzed, a supporting video in .avi format was created (see Supplementary Materials) with the same CellTracker program, which provides visual information of cell–material interactions. Trajectory processing with MATLAB helped to obtain different kinematic parameters, such as total and average length travelled by the cells, maximum and average distance travelled and maximum and average cell speed. Significance of differences in parameters was tested by computing the P value using unpaired t-test analysis.

## 3. Results

The described design, manufacturing and replication processes lead to polyacrylamide gel cell culture substrates with design-controlled topographies. Figure 1 shows the computer-aided design of microstructured surfaces, including both positive and negative alternatives: In Figure 1a a microtextured vascular-like region upon a plane can be seen, while Figure 1b presents a planar vascular-like region surrounded by microtexture. Figure 2 briefly schematizes how the designs are materialized: Figure 2a presents direct laser written microstructures as master models and Figure 2b includes detailed views of vascular-like microstructures. Figure 2c presents the mold insert obtained after electroplating and mounted in the hot-embossing tool, while Figure 2d shows a pattern transferred to the polyacrylamide gel. The checkerboard-like pattern of Figure 2a, obtained after a matrix-based replication of the designs of Figure 1, is aimed at higher throughput and more systematic testing: several experiments may be performed in a single culture substrate.

It is important to put forward that the microbumps of the original CAD design, after manufacturing and pattern transferring by soft-lithography, lead to microwells upon the polyacrylamide gel surfaces, which prove adequate for anchoring cells to desired positions. The overall volume of the microwells is similar to that of cells being cultured. Planar areas and regions with microwells can be defined, thanks to soft-lithography, upon the surfaces of polyacrylamide substrates for a wide set of applications.

The proposed combination of techniques and processes allow for the creation of microstructures with overall sizes in the order of magnitude of common cell types (e.g., 10 × 10 × 10 μm^3^). A closer look at the “H-like” vascular patterns helps to realize that the different branches of the “H” vascular patterns have different widths:

The central channels have a width of 30 µm for letting groups of three cells interact, the lateral channels have a width of 20 µm for trapping cells in couples and the more external channels of the “H” figures have a width of 10 µm, within which only a row of single cells can be arranged. In this way, single cells, couples and triplets can be arranged for studying cell-cell interations.

The gradients of width in the template are included with two main motivations: 

On the one side, changing their thickness in a methodic way allows to check the precision and viability of these manufacturing processes for achieving multi-scale and complex surface topographies, which is confirmed. On the other hand, it may support the topography-guided organization of cells, forming rows or columns, duplets and triplets, for a wide set of potential uses in the lab- and organ-on-a-chip field.

Although minor manufacturing flaws appear in some of the direct laser written and electroplated boxes of the checkerboard template, it is important to mention that most boxes are perfectly manufactured and show exactly the same morphology of the designs shown in Figure 1a,b and of the manufactured images of Figure 2a,b.

In some cases a written row of microbumps is displaced or a couple of microbumps are lost, without affecting overall performance. In any case, most “H-like” microstructures or patterns are similar. In addition, the electroplated insert used for hot-embossing is able to create a test series of 50 replicas without showing any damage. Besides, the thermoplastic replicas can be used for soft-lithography purposes, without suffering any scratch or harm due to the softer nature of the polyacrylamide gel. Consequently, productivity can increase exponentially, if hot-embossed series are used several times for pattern transferring.

**Figure 3:**
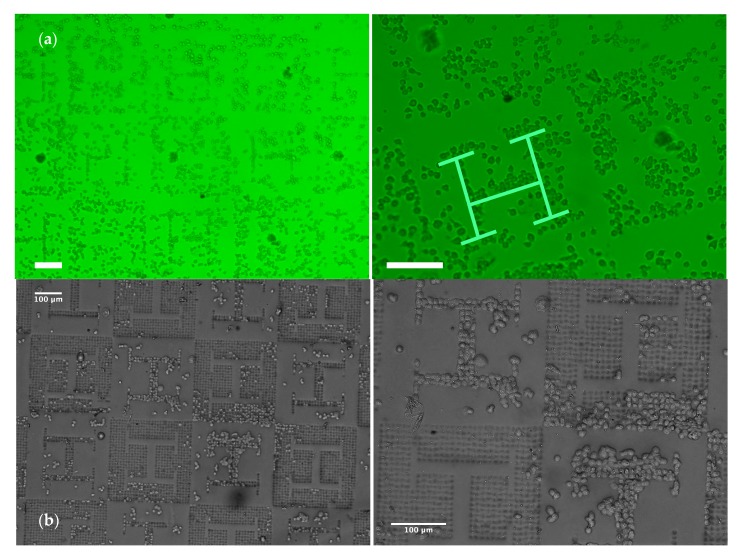
Results from *Dictyostelium discoideum* culture processes, performed upon different replicas of the polyacrylamide substrates obtained by soft-lithography: (**a**) MEIJI TC5400 optical microscope with phase contrast, (**b**) Leica EZ4HD stereoscopic microscope. Visual inspection shows how cells are organized due to the presence of topographical transitions. H-like cell patterns can be perceived in regions, where the transferred microwells create a vascular pattern; while larger cell clusters can be seen in regions, in which microwells surround planar vascular-like zones. White scale bars = 100 µm.

**Figure 4:**
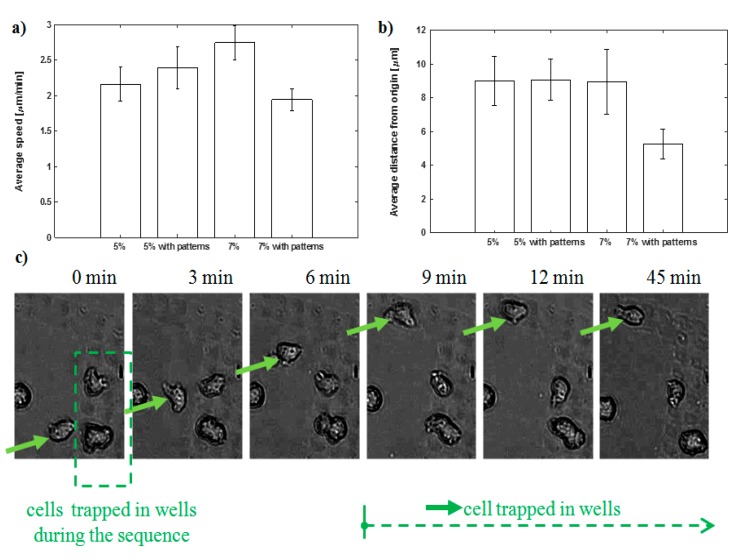
Influence of gel patterning and stiffness on amoeboid cell behavior: (**a**) Average cell speed; (**b**) average distance travelled by the cells from the origin to their final position during an experiment of 45 min; (**c**) representative sequence of images showing the movement and trapping of one cell in the wells of the gel.

Once manufacturability is confirmed, cell culture experiments are carried out following the methods described in Section 2.4 and Section 2.5. As shown in the attached Supplementary Video (Video S1) and presented in the summary images from Figure 3 and Figure 4, *Dictyostelium discoideum* amoeboid cells, cultured upon the polyacrylamide substrates, interact with the surface showing an amoeboid motion with a speed of around 2–2.5 µm/min (Figure 4a), typically until they get trapped by the microwells generated upon the substrate, where they remain and rarely move away (Figure 4c). Figure 3a,b (and their zoomed-in views) shows that the surface pattern leads to non-uniform distributions of the cell population upon the surface and “H-like” aggregations can be perceived (as highlighted in pale green in the zoomed-in view of Figure 3a). In the regions with planar “H-like” patterns surrounded by microwells, trapped cells leaving an empty “vascular” region within can be also appreciated. In accordance with the width of vascular patterns, single-cell rows, duplets, triplets and clusters of cells can be appreciated.

The cell culture processes performed verify the possibility of influencing cell populations cultured upon polyacrylamide hydrogels just by using controlled gradients and transitions of surface topography. This combination of processes and technologies may be also applied to structuring other hydrogels and to the mass-production of hydrogel templates. Potential for several types of cell culture processes and for the development of lab- and organ-on-a-chip devices, in which cell co-cultures may help to study disease in more biomimetic conditions than using conventional Petri dishes.

The employment of amoeboid cells, especially *Dictyostelium discoideum*, whose easy genetic manipulation and social behavior makes them a nice model for a wide set of biological and biophysical problems, is a first approach towards relevant biological and medical applications of the processes and technologies presented. These may open new fields of application for hydrogels.

Cell visualization and tracking following the processes described in Section 2.5 lead to the results summarized in Figure 4. The influence of gel patterning and stiffness on amoeboid cell behavior is presented, including data on average cell speed (4a) and average distance travelled by cells from the origin to the final position in the sequence (4b): Cells moving upon planar regions and upon textured regions with presence of microwells are compared. The influence of substrate stiffness is presented, as 5% and 7% polyacrylamide hydrogels are employed for diverse experiments. The differences in distance from origin are only significant for the patterned regions of the stiffer gels. The differences in average speed are again not significant for three of the cases (P ≥ 0.10 for a concentration of 5% and for 7% without patterns), while for a concentration of 7% the average speed is slightly lower on the gels with patterns (P = 0.01). This difference, which would indicate a lower average speed on more rigid substrates, is in agreement with the observed larger stability of cell-substrate interactions for rigid substrates in the case of some cells [31]. It is also possible that some stick-slip phenomena may be present and that stiffer substrates can promote more rapid oscillations of the cells (see Video S1), even if the travelled distance remains unaffected.

However, further studies will be required to more adequately understand these effects in our gels and the influence of gel stiffness and surface topography on cell movement; now that cell trapping and the possibility of controlling cell populations upon hydrogel biointerfaces, by means of microtextured wells and channels, have been verified. In any case, the differences found are significant just for the patterned regions of the stiffer gel, in which case an increase of speed is also consistent with a larger distance travelled. This interesting synergy between stiffness and texture for increasing cell speed and travelled distance will be further studied employed in future research, towards application of the presented processes.

## 4. Discussion: Potentials, Limitations and Continuation Proposals

In connection with the fourth biotechnological revolution, controlling cell populations upon biointerfaces is of extreme relevance, as this may play a key role for developing the “biofactories of the future” for sustainable and efficient production processes [32]. The presented processes and techniques, applied to microstructuring the surfaces of polyacrylamide hydrogels and influencing the distribution of cultured amoeboid cells, may be also applied to other microorganisms such as yeast, fungi and cyanobacteria for industrial production and biological processes (i.e., fermentation, oxygenic photosynthesis, among others) [33,34].

The arrest effect of microwells is very interesting and a plausible explanation is that it is due to gravity: The density of cells, slightly larger than water [35], would bring them downwards and into the wells. It was previously shown that *Dictyostelium* cells can exert a force large enough to compensate gravity on a vertical surface [36], and in fact in our experiments some of the cells could exit from wells after being trapped. However, the erratic movement of these amoebas is reasonably affected by the force. An alternative explanation could be related to the contribution of different properties of the hydrogel surface in the wells and to surface tension effects, increased in the microwells, due to their greater surface/volume ratio, as compared with planar regions. Probably both effects synergize in a positive way. In any case, the use of micropatterned surfaces proves of interest for biophysical studies, due to the possibility of using gravity, surface tension and orientation of surfaces to control the effective direction of the force acting on the cell.

Regarding biomedical applications, future studies may also deal with culture processes using animal and human cells and co-culturing different cell types, for which polyacrylamide substrates are also expected to provide an interesting culture environment. 

Towards real 3D cell culture microenvironments, it may be also interesting to combine a couple of microtextured surfaces or chips, culturing two different cell types upon them and placing the chips face-to-face (with the parenchymal regions facing the vascular zones). In such a way, it may be possible to achieve sandwiched microenvironments to study other cell–material interactions [37]. Furthermore, the defined transitions of planar and microtextured regions, can be applied to the creation of microfluidic devices “on chips”, in which different zones may be defined just by design-controlled surface texturing upon a single functional layer or surface.

In the authors’ opinion, the possibility of controlling cell populations upon hydrogel templates by resorting only to controlled surface patterns can support the development of a new generation of labs- and organs-on-chips, in which the number of components (layers, tubes, membranes, clamping elements) may be reduced. 

Once cells are controlled just by surface texturing and by employing special biointerfaces, as the ones described here, usability of labs- and organs-on-chips may be also enhanced. First of all pipetting processes may be simplified, if cells stirred upon the surface arrange themselves just by the effect of topography. In addition, visualization procedures will be easier, when a single and open textured biointerface replaces a multi-layer microfluidic system. In some cases the employment of additional surface functionalizations may be needed, especially if other processes and materials are employed, as shown in recent research by our team [38].

Different types of labs- and organs-on-chips may be developed with the support of the presented processes. The geometries presented in this study as conceptual validation are inspired by vascular bifurcations. If endothelial cells are trapped in wells arranged forming a vascular network and other types of parenchymal cells (such as hepatocytes or neurons and glial cells) are cultured in their surroundings, it may be possible to emulate some complex physiological interactions in organs, including liver or brain, to cite two examples. With other configurations, tumors-on-chips may be developed: In these microsystems, microtextures may be employed for trapping healthy cells in biomimetic arrangements and microwells may be used for fixing tumoral spheroids to desired positions. More complex configurations may be achieved by resorting to the aforementioned sandwiched microenvironments and by combining soft hydrogel templates with more rigid biomaterials, so as to emulate the stiffness transitions present in the interfaces between our soft and hard tissues.

Regarding industrialization of the presented processes, the potential toxicity of polyacrylamide [39] should be considered and managed. Apart from that, manufacturing technically relevant samples with areas of some cm^2^ is still a limitation for processing chains starting with a two-photon polymerization step. Its accuracy is outstanding and remarkable for conceptual proofs of different micro and nanosystems, but the writing speed and attainable part size are still limited for mass production. A possible approach for enhanced productivity may rely on more complex master models (e.g., cylinders), which may be applied to roll-to-roll processing of polymeric substrates or templates. Such templates, in turn, may be used for pattern transferring by soft-lithographic processes to polymeric hydrogels.

## 5. Conclusions

In this research, a novel strategy for the surface processing of polyacrylamide hydrogels, based on combining soft-lithography with microstructured templates, has been described, demonstrated and analyzed. Summarizing, the process involves the following steps: 

(1) Design of textured surfaces and creation of templates by 3D direct laser writing; (2) electroplating for obtaining production tools; (3) gel synthesis and processing by soft lithography using the production tools; and (4) cell culture for analyzing the effects of surface topography on cell populations.

The novelty of the process relies on the templates used, whose multi-scale microstructures are designed by means of 3D computational modeling and manufactured as master models by direct laser writing of photopolymers. 

Subsequently, thin-film metallic deposition and electroplating transforms the master models in mold inserts, which are employed for systematic cost- and time-effective replication of microstructured templates employing hot-embossing (or compression molding) upon thermoplastics. The templates obtained through hot-embossing of PMMA are transferred by soft-lithography, with remarkable level of detail, to the polyacrylamide hydrogels.

The conceived process has been demonstrated by patterning polyacrylamide substrates with a set of vascular-like and parenchymal-like regions, defined by transitions of topography, which are conceived as potential functional layers or biointerfaces of lab- and organ-on-a-chip devices for controlling cell populations. Final culture of amoeboid cells, whose dynamics has been shown affected by the polyacrylamide patterns, has provided a preliminary validation of the described strategy and helped to discuss its potentials, towards more complex and biomimetic co-culture systems and cell cultivation set-ups. The possibility of creating controlled cell patterns and cell clusters, just by trapping amoeboid cells with the support of the microwells generated by soft-lithography upon the polyacrylamide gel surface, has been demonstrated.

## Figures and Tables

**Table 1:** Used materials for polyacrylamide gel synthesis.

Material	Condition	Supplier
NaOH	98%	Panreac
APTES ((3-aminopropyl)triethoxysilane)	99%	Sigma-Aldrich
Glutaraldehyde (GA)	25% in H_2_O	Sigma-Aldrich
PBS (phosphate-buffered saline)	Solution 1× and 10×	Sigma-Aldrich
AB-mide (acrylamide/bis-acrylamide)	Solution 40%	Sigma-Aldrich
APS (ammonium persulfate)	99%	Acros Organics
TEMED (tetramethylethylenediamine)	99%	Sigma-Aldrich

**Table 2:** Reagents for polyacrylamide gel synthesis.

Gel	Acrylamide/Bis-Acrylamide Solution	Ammonium Persulfate	TEMED	Distilled Water
5%	250 µL	17 µL	5 µL	1750 µL
7%	350 µL	23 µL	7 µL	1650 µL

**Table 3:** Chemical composition of culture medium HL5-C.

Formula	g/L
Peptone	5
Yeast extract	5
Tryptone	5
KH_2_PO_4_	1.2
Na_2_HPO_4_	0.35
Glucose	10

## Supplementary Materials

The following are available online at https://www.mdpi.com/1996-1944/13/7/1586/s1, Video S1: Attached videos show the amoeboid cells moving upon and interacting with the textured hydrogel surfaces. Anchorage to microwells can be appreciated.
